# Pressure Ulcer Associated with Testicular Prosthesis as a Rare Cause of Spinal Epidural Abscess

**DOI:** 10.1155/2019/9090462

**Published:** 2019-08-14

**Authors:** Amulya Prakash, Rishi Raj, Aasems Jacob, Douglas Ross

**Affiliations:** ^1^Monmouth Medical Center, Long Branch, NJ, USA; ^2^University of Kentucky, Lexington, KY, USA

## Abstract

Spinal epidural abscess is a neurologic emergency with a potential complication to the spinal cord such as paralysis. Frequently, it has a nonspecific initial presentation such as neck or back pain, and hence there is a delay in diagnosis. We present the case of a 60-year-old Caucasian male who presented to emergency room with one week of numbness and weakness of all four extremities. Neurological examination showed variable quadriparesis. Urgent MRI of spine with contrast revealed epidural abscess in the cervical region C4–C6 with resultant cord compression, the underlying etiology for hematogenous spread of infection being pressure ulcer associated with testicular prosthesis. Urgent neurosurgical intervention was done to achieve spinal cord decompression. Both blood and pus cultures were positive for *Streptococcus intermedius*, requiring prolonged administration of intravenous antibiotics. Clinical outcome was encouraging with progressive gain in motor and sensory function. Spinal epidural abscess is a rare diagnosis; hence, clinicians should have a high index of suspicion for timely diagnosis.

## 1. Introduction

Spinal epidural abscess is a rare infectious process of the spine. Early diagnosis and management are pivotal in preventing irreversible neurological deficits; however, the outcome is multifactorial depending on patient's age, duration of motor weakness, septic presentation, and location of abscess [1]. In nonspecific cases of neck and back pain, identification of risk factors and comorbidities is important for risk stratification and to raise the index of clinical suspicion which will aid in prompt diagnosis and improved outcomes. We present the case of a 60-year-old gentleman who presented to his primary care physician's office with a complaint of neck pain without any sign of infection or any neurologic deficit. The patient was initially managed with acetaminophen, but his symptom of neck pain worsened, and later on he developed quadriplegia.

The case presented below highlights one of the rare and catastrophic differential diagnoses of neck and back pain. Our patient had a very unusual nidus of infection which was indeed difficult to suspect. This case is unusual also in regard to the location of pathology as the patient had epidural abscess in the cervical region which is very rare; the organism isolated was *Streptococcus intermedius* which is uncommon.

## 2. Case Presentation

A 60-year-old male presented to the emergency room with a complaint of weakness and numbness of all extremities from last three days. One week prior to the presentation, the patient visited his primary care physician with a complaint of neck pain and was sent home on acetaminophen with a possible diagnosis of musculoskeletal pain. His symptoms worsened and rapidly progressed to new onset weakness of all extremities and numbness all over below the level of neck, associated with bowel and bladder incontinence. The patient has a past medical history of diabetes mellitus, thalamic stroke three years ago, and a prosthetic left testis because of undescended left testis. Previously, the patient had excision of the skin and a sinus tract of the left scrotal wall; however, there is no evidence of active infection.

At the time of presentation, vital signs were noted to be BP of 140/85 mm of Hg, pulse rate of 85 beats per minute, respiratory rate of 18, temperature of 99.7°F, and pulse oximetry of 97% on room air. On examination, appearance was nontoxic, endorsing a low-grade fever. Cardiopulmonary examination was benign. Cranial nerve examination was unremarkable, and neck movement was unrestricted. Upper extremities had a strength of 3/5 in the right and 2/5 in the left, and lower extremities had a strength of 2/5 in the left and 3/5 in the right. Sensation to fine touch and pin prick was grossly reduced below neck; however, pain, pressure, and vibration sense were intact. Reflexes were intact; Babinski's sign was equivocal. Other significant finding in physical examination was pressure ulcer over left scrotal skin from prostheses but no pus collection or drainage.

**Figure 1:**
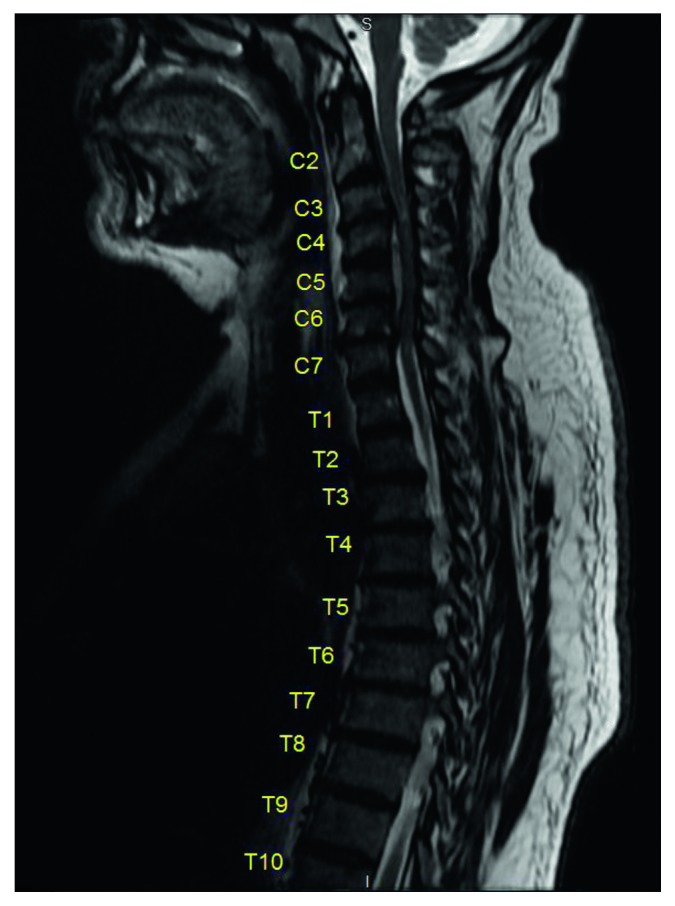
MRI with contrast of spine shows peripherally enhancing, approximately 1.2 × 4.6 cm, T2 hyperintensity/T1-hypointense collection that is located along the posterior vertebral bodies C4–C6.

Investigations done at the time of admission revealed high inflammatory activity with a CRP level as high as 133.3 mg/L and leucocytosis with a cell count of 17,000 cells/mL. Blood cultures were drawn, and empiric broad-spectrum antibiotics were initiated. For a new onset quadriparesis with neck pain, an MRI of whole spine with and without contrast (Figure 1) was performed emergently which revealed a collection that was located along the posterior aspect of vertebral bodies C4–C6, suggestive of epidural abscess with severe cord compression and focal T2 hyperintensity at the C3-C4 level, concerning cord edema. There were no evident cervical infiltrations.

Multidisciplinary care was initiated for this patient. Neurosurgery was contacted for emergent spinal decompression. The pus was drained, and C3-C4 fusion with plating was done. Our patient was started empirically on cefepime and vancomycin which were tailored as per culture and sensitivity findings; the antibiotics were continued for a total duration of 42 days. Both blood and pus culture from the spine grew *Streptococcus intermedius*, susceptible to ceftriaxone. Testicular prosthesis gross examination was normal. The culture obtained from the pressure ulcer site over scrotum grew mixed flora including *Streptococcus intermedius*. Testicular prosthesis was removed with excision of affected scrotal wall. Our patient had a remarkable recovery, and one-year follow-up on the patient shows no residual neurologic symptom.

## 3. Case Discussion

Spinal epidural abscess (SEA) is a rare diagnosis with less than 2 cases per 10,000 hospital admissions [2]. However, there has been a recent increase in incidence. The mortality from nontuberculous SEA remains at around 13–16% [3]. Long-term complications arose from sepsis, prolonged immobility, and neurological deficits due to delay in diagnosis. The biggest challenge in the diagnosis is nonspecific and subacute presentation, usually presenting as a case of neck or back pain, often being initially treated with analgesics and muscle relaxants and leading to a delay in diagnosis till manifestation of neurological symptoms. The classical triad of fever, back pain, and neurological symptoms has been reported in only 10–15% of the cases.

Our patient had a rare presentation of SEA as it involved the cervical spine. In most cases of SEA, predominance of abscess is in thoracic and lumbosacral areas. Another interesting finding was isolation of *Streptococcus intermedius* from abscess. The most common organism is *Staphylococcus aureus*, presenting in about 70% of all cases of SEA, followed by *Streptococcus* species which accounts for only 7% of the cases [4]. Among *Streptococcus* species, *S. constellatus* and *S. anginosus* have been isolated from a visceral abscess far more frequently than *S. intermedius* [5]. Interestingly, fewer cases of brain and liver abscesses caused by *S. intermedius* have been reported, but cases of SEA are even more rare [6].

Identification of risk factors can help stratify and raise the index of clinical suspicion leading to early diagnosis. Common risk factors are neurosurgical interventions, epidural cannulations [7], IV drug abuse [8], and diabetes mellitus or infection at another site such as infective endocarditis [9]. However, cases have been reported without predisposing risk factors, as a case of spontaneous abscess [10]. In this case, we suspect the pressure necrosis of scrotal wall from prosthetic testes was the source of blood stream infection and led to hematogenous spread of infection to the spine.

Diagnosis is most of the times confounded by nonspecific symptoms and hence warrants early utilization of diagnostic modalities. MRI with gadolinium contrast has sensitivity and specificity higher than 90% [11]. Liquid and granulation tissue can both be visualized with T1-weighted and contrast-enhanced images, respectively. Lumbar puncture is not required for diagnosis, should rather be avoided because of potential spread of bacteria to subarachnoid space leading to meningitis.

Management of choice is surgical decompressive laminectomy and washout, followed by a prolonged antibiotic course of at least 6 weeks [12], which is tailored as per sensitivity of the cultured organism from pus. Medical management with prolonged antibiotic therapy alone and/or decompression with percutaneous drainage have been successfully tried [13, 14]. There has been a substantial trend toward treating neurologically intact patients with medical management. Nevertheless, medical therapy fails in a fair number of cases involving patients with specific risk factors, and the patients with these risk factors should be closely observed in consideration for surgery [15]. Review of multiple analysis and case series proves early decompression surgery with antibiotics has a better outcome and fewer chances of neurological complications [16].

In conclusion, early diagnosis and intervention is the key to reduce mortality and neurological complications. Preoperative neurological status is the best predictor of the final neurological outcome, but it is difficult to anticipate the frequency and extent of neurological deficits. Despite aggressive diagnosis and management, the mortality still remains around 13–16%. The biggest challenge for clinicians remains the early diagnosis and intervention.
